# Patient and provider perspectives of pluralistic primary care services in urban Bangladesh: a qualitative study

**DOI:** 10.1186/s12913-026-14106-z

**Published:** 2026-02-14

**Authors:** Nabila Binth Jahan, Deepa Barua, Helen Elsey, Fatema Kashfi, Rumana Huque, Mahua Das

**Affiliations:** 1 ARK Foundation, Suite A-1, C-3 & C-4, House # 6, Road # 109, Gulshan 2, Dhaka, 1212 Bangladesh; 2 Global Public Health, Hull and York Medical School, University of York, Heslington, York, YO10 5DD UK; 3 Nuffield Centre for International Health and Development, University of Leeds, Leeds, LS2 9JT UK

**Keywords:** Primary health care, Urban poor, NGO clinics, Government outdoor dispensaries, Non-communicable diseases, Bangladesh

## Abstract

**Background:**

Rapid urbanisation and the growing prevalence of non-communicable diseases (NCDs) present significant challenges for low- and middle-income countries (LMICs). Among these, Dhaka, the densely populated capital of Bangladesh, experiences these pressures more intensely than most. As a result, the city's public primary health care (PHC) services are struggling to meet the increasing health demands of its urban poor. This study, therefore, examines healthcare access and utilisation in Dhaka's urban slums, giving special attention to the contrasting perceptions of service delivery held by healthcare providers and patients regarding NCD care.

**Methods:**

We used a qualitative case study approach, purposively sampling four NGO clinics and two Government Outdoor Dispensaries (GoDs) as our cases, all of which are accessible to individuals of diverse age groups, genders, and socio-economic backgrounds. For each case, we conducted in-depth interviews with male, female, and hijra patients within the catchment areas. The data collection process took place between December 2022 to August 2023, following the Consolidated Criteria for Reporting Qualitative Research (COREQ) guideline.

**Results:**

Results from this study identified key factors influencing healthcare-seeking behaviour at PHC centres for NCDs. These included: (1) Costs and perceptions of cost, (2) Availability and quality of NCD medicines and opening times of facilities, (3) Behaviour of providers and patients, particularly with socially stigmatised groups, (4) Perception of PHC focus on Maternity and Neonatal Child Health (MNCH), and (5) Patient perceptions of NCDs.

**Conclusions:**

Despite the increasing prevalence of NCDs, patients face multiple barriers to accessing PHC services. These barriers stem not only from limitations in the services provided but also from patients' perceptions of both NCDs and primary care itself, which significantly influence their health-seeking behaviour. This study advances understanding of barriers to PHC utilisation in Dhaka by revealing how men and hijras are excluded, how restricted facility hours limit working-class men's access, and how social networks can help reduce exclusion. While global frameworks offer valuable guidance, context-specific strategies such as public-private collaboration, inclusive service design and community engagement are necessary to address persistent inequities in PHC access in Dhaka City.

**Supplementary Information:**

The online version contains supplementary material available at 10.1186/s12913-026-14106-z.

## Background

Access to primary health care (PHC) in Bangladesh remains critical for reducing morbidity and mortality, especially given the rising burden of non-communicable diseases (NCDs) [1]. Factors influencing people's choice of PHC facilities vary across different contexts and are influenced by complex demand and supply-side characteristics [2]. The impact of rapid urbanisation, along with the progression of demographic and epidemiological transition, has led to sedentary lifestyles, unhealthy diets, and increased stress, driving a rise in NCDs such as hypertension, diabetes, and cardiovascular diseases [3]. Bangladesh, a country that has seen the urban growth rate rise from 7.9% in 1971 to 38.18% in 2020 [4], has a rising NCD prevalence that causes 580,000 deaths annually, accounting for 67% of total deaths [5].

Addressing the challenge of the growing disease burden caused by NCDs in a rapidly urbanising context is difficult, partly because the NCD control budget is relatively small [6], but also due to a limited focus on primary care within urban areas. This is despite the World Health Organization's (WHO) recommendation to allocate a substantial portion of the budget to PHC to achieve UHC, Bangladesh dedicates only 2.36% of its gross domestic product (GDP) [7]. Although policy initiatives such as the 4th Health, Population and Nutrition Sector Program (HPNSP) and the Health Strategy under the 7th Five-Year Plan aim to strengthen NCD control, overall investment in this area remains limited, accounting for only 4.2% of the total budget [8, 9].

To address the growing burden of NCDs, the government developed a national NCD plan in 2011 and from 2012, established NCD service corners in sub-national hospitals. While these corners are operational in rural areas, urban public primary care centres currently lack such dedicated NCD services [10]. The governance arrangements for urban primary care go some way to explain this urban–rural difference. In rural areas, public primary care falls under the Ministry of Health and Family Welfare (MoHFW). However, in urban areas, the limited availability of public primary care facilities has resulted in a mixed service delivery system, where primary care is provided through Government Outdoor Dispensaries and NGO operated facilities. This has led to complex governance arrangements with NGO-operated PHC facilities falling under Ministry of Local Government, Rural Development and Co-operatives (MoLGRDC) and Government Outdoor Dispensaries (GoDs) under the Civil Surgeon (CS) Office of the MoHFW [11, 12]. This divided governance framework often results in fragmented service delivery, weak coordination, and blurred lines of accountability, which influence service quality, referral pathways, and provider behaviour [13].

Nevertheless, patients' healthcare-seeking decisions are shaped by factors that extend beyond these government initiatives. Among these, financial burden remains one of the most significant barriers, with out-of-pocket expenditures accounting for 72.99% of per capita health spending [7]. Other key factors influencing patients' choices of healthcare facilities include opening hours, patient satisfaction, travel distance, waiting time, illness duration, care quality, and geographic and financial barriers [14–16]. Additionally, satisfaction with healthcare providers' plays a crucial role in determining whether patients will utilise PHC services [17]. Previous studies have consistently demonstrated significant gender disparities in accessing PHC, with women more frequently visiting PHC facilities than men, particularly for preventive care and chronic conditions [18]. In addition, marginalised groups, such as the third gender *(hijra)* community in Bangladesh encounter pervasive barriers to healthcare access, including stigma and discrimination, lack of recognition within hospital procedures, and inadequate infrastructure such as shared wards that do not accommodate gender-diverse patients [19]. However, research on this is limited, yet it is clear that they face substantial barriers due to stigma and discrimination. These barriers not only affect their access to healthcare but also exacerbate existing health disparities. This lack of research on their experiences highlights a critical gap in understanding how these barriers impact their access to primary healthcare services.

### Objectives

To address these gaps, this study aims:

- To explore the facilitators and barriers to accessing and delivering PHC services in Dhaka city, from both patient and provider perspectives.
- To explore the impact of out-of-pocket expenditures, mismatched service hours, and social stigma on healthcare-seeking behaviour.

## Methods

### Study design

This study complies with the Consolidated Criteria for Reporting Qualitative Research (COREQ) guidelines [20]. A case study approach [21, 22] was used to understand how urban PHC facilities are perceived and used by those in their catchment areas within the context of Bangladesh. We used a collective instrumental approach [21] by studying multiple cases from the Dhaka North City Corporation (DNCC) simultaneously. This approach was chosen because it allows for both within-case and cross-case analysis, facilitating the identification of shared patterns and context-specific differences across diverse institutional and socio-economic settings [21]. Given the pluralistic structure of urban health systems in Bangladesh, where NGO clinics and GoDs operate under overlapping mandates, this approach was particularly suited to revealing how contextual and organisational factors collectively shape access to and use of PHC services. Within each case study site, we conducted observations and interviews with both healthcare providers and patients residing in the surrounding areas.

### Selection of the case study sites

We selected DNCC as our study area as it has the largest population of all 12 city corporations in Bangladesh [23]. Within DNCC, 32 NGO clinics are providing services through Primary Health Care Centres (PHCC), Comprehensive Reproductive Health Care Centres (CRHCC), and Satellite Clinics under the MoLGRDC. In contrast, nine GoDs are currently operating in DNCC under the MoHFW, classified into four categories: (i) general GoD: accessible to all types of patients; (ii) school health clinics; (iii) maternity clinics, and (iv) clinics situated within other government facilities, which are more accessible to government employees and their family members.

Of the nine government dispensaries and 32 NGO clinics operating within DNCC, we purposively selected four NGO clinics under the Urban Primary Health Care Service Delivery Project (UPHCSDP) and two GoDs for our case study. All selected facilities shared similar operational characteristics: NGO clinics function under the UPHCSDP of the MoLGRDC, while GoDs operate under the Civil Surgeon's Office of the MoHFW, following comparable operational modalities and structural standards. Selection was guided by the larger number and wider geographic coverage of NGO clinics, the limited availability and accessibility of GoDs (two of which are located within government compounds and not open to the public), the willingness of facilities to participate, the availability of administrative support, robust infrastructure, high patient volume, adequate staffing (at least one physician, one medical assistant, and one pharmacist) (See Table 1), and strategic location near urban poor neighbourhoods, ensuring comprehensive PHC services for all patient groups.

**Table 1:** The cases: NGO clinics and government outdoor dispensaries (GoDs)

The cases: primary healthcare centres	Case study number	Case study characteristics	Number of interviews conducted with patients from the case study area
NGO Clinics	Case Study- 1	− Established in 1998 under the UPHCDP and supervised by the MoLGRDC, provides 24-hour indoor and outdoor care for common illnesses and maternity care − Located in a four-story building, and has 11–14 rooms and 16 healthcare providers − Serves patients from nearby informal settlements and catchment areas − Overseen by a female in charge, attracting more female patients − Lab services- minor surgeries and C-sections are available. Diagnostic tools- blood pressure machines and glucometers are also available, but lack NCD-related medicines	Female − 1
			Male − 1
			Hijra − 3
	Case Study- 2	− Established in 1998 under the UPHCDP and supervised by the MoLGRDC, provides 9 am to 4 pm outdoor care similar to other centres − Located in a two-story building, and has 11–14 rooms and 16 healthcare providers. A restroom has been converted into a counselling area, and stairs serve as a breastfeeding space − Serves patients from nearby informal settlements and catchment areas − Overseen by a male in charge, attracting more male patients − Lab facilities, including a blood pressure machine and glucometer, are available but lack NCD-related medicines	Female − 1
			Male − 3
			Hijra − 2
	Case Study- 3	− Established in 1998 under the UPHCDP and supervised by the MoLGRDC, provides 9 am to 4 pm outdoor care similar to other centres − Provides services on a single floor within a four-story building and has nine rooms and 16 service providers − Primarily serves patients from its catchment areas and nearby informal settlements − Overseen by a female in charge, attracting more female patients − Lab facilities are available, and minor surgeries and C-sections are available on demand. A blood pressure machine and glucometer are available, but lack NCD-related medicines	Female − 1
			Male − 1
			Hijra − 3
	Case Study- 4	− Established in 1998 under the UPHCDP and supervised by the MoLGRDC, provides 9 am to 4 pm outdoor care similar to other centres − In a four-storey building, providing PHC services on the ground floor only with well-equipped infrastructure, with 11 rooms, and 16 healthcare providers. Other floors are being used for CRHCC − Primarily serves patients from its catchment area − Overseen by a female in charge, attracting more female patients − Lab services- minor surgeries and C-sections are available. Diagnostic tools- blood pressure machines and glucometers are also available, but lack NCD-related medicines	Female − 1
			Male − 1
			Hijra − 2
Government Outdoor Dispensaries (GoD)	Case Study- 5	− Established in 2000, operating under the CS Office of the MoHFW, provides 9 am to 2 pm outdoor care − Functions in a one-storey building with four rooms and six healthcare providers − Led by a female in charge, the facility serves patients from diverse social backgrounds and genders, with a high patient turnout − Don't have any catchment areas, often overcrowded with patients from surrounding areas − No lab facilities available, but has Blood Pressure machine and glucometer, and NCD-related medicines. Well-equipped for NCD care, attracting more NCD patients	Female − 3
			Male − 2
	Case Study- 6	− Established in 2000, operating under the CS Office of the MoHFW, provides 9 am to 2 pm outdoor care − Single-storey building with eight rooms and six healthcare providers − Led by a male in charge, the facility accommodates patients from diverse social backgrounds and genders, experiencing a high patient volume − Don't have any catchment areas, often overcrowded with patients from surrounding areas − No lab facilities available, but has blood pressure machine and glucometer, and NCD-related medicines. Well-equipped for NCD care, attracting more NCD patients	Female − 3
			Male − 2

### Sampling of participants within case study sites

#### Sampling of patient

We selected male, female, and hijra community members within the catchment areas of our case study sites. Purposive sampling was used for males and females, while a snowball sampling technique was employed for hijra patients due to their limited visibility around PHC facilities. Initially, we approached patients face to face *(n = 35)*, and selected 10 males, 10 females, and 10 hijra patients *(n = 30)*, regardless of their NCD status and from diverse socio-economic backgrounds (See Table 2) to ensure a comprehensive representation of experiences and perceptions related to PHC utilisation among both regular service users and community members residing within, and familiar with the facilities catchment areas. Two hijra patients and three male patients declined to participate in the study due to time constraints and lack of interest.

**Table 2:** Participant characteristics

Case	Age	Gender	Level of Education	Family Income	Case Study Setting	NCDs
PT_F_G_16	Above 40 years	Female	Never attended	Above 20,000 TK	Case Study Setting − 6	Diabetes
PT_F_G_17	Above 40 years	Female	Class 1–5	Not Reported	Case Study Setting − 6	Hypertension
PT_F_N_10	36 to 40 years	Female	Never attended	5,000 to 10,000 TK	Case Study Setting − 1	Hypertension
PT_F_N_25	Not Reported	Female	Class 1–5	10,000 to 15,000 TK	Case Study Setting − 2	Hypertension
PT_F_N_26	18 to 25 years	Female	Class 6–8	10,000 to 15,000 TK	Case Study Setting − 3	Not Reported
PT_F_G_15	Above 40 years	Female	Not Reported	10,000 to 15,000 TK	Case Study Setting − 5	Not Reported
PT_F_N_28	Not Reported	Female	Class 6–8	15,000–20,000 TK	Case Study Setting − 4	Diabetes
PT_F_G_07	30 to 35 years	Female	Class 1–5	15,000–20,000 TK	Case Study Setting − 6	Not Reported
PT_F_G_08	Above 40 years	Female	Never attended	10,000 to 15,000 TK	Case Study Setting − 5	Hypertension
PT_F_G_09	36 to 40 years	Female	Not Reported	10,000 to 15,000 TK	Case Study Setting − 5	Diabetes
PT_M_G_01	30 to 35 years	Male	Not Reported	Above 20,000 TK	Case Study Setting − 6	Not Reported
PT_M_G_12	Above 40 years	Male	Class 1–5	10,000 to 15,000 TK	Case Study Setting − 5	Not Reported
PT_F_G_19	Above 40 years	Male	Not Reported	10,000 to 15,000 TK	Case Study Setting − 5	Hypertension
PT_M_G_02	Above 40 years	Male	Class 1–5	Above 20,000 TK	Case Study Setting − 6	Hypertension
PT_M_N_05	30 to 35 years	Male	Class 1–5	Above 20,000 TK	Case Study Setting − 4	Diabetes
PT_M_N_03	36 to 40 years	Male	Never attended	5,000 to 10,000 TK	Case Study Setting − 2	Not Reported
PT_M_N_18	Above 40 years	Male	Never attended	15,000–20,000 TK	Case Study Setting − 2	Hypertension
PT_M_N_27	Not Reported	Male	Not Reported	5,000 to 10,000 TK	Case Study Setting − 3	Not Reported
PT_M_N_29	30 to 35 years	Male	Never attended	15,000–20,000 TK	Case Study Setting − 2	Hypertension
PT_M_N_30	Above 40 years	Male	Class 1–5	15,000–20,000 TK	Case Study Setting − 1	Diabetes
PT_T_N_06	36 to 40 years	Hijra	Not Reported	15,000–20,000 TK	Case Study Setting − 1	Diabetes
PT_T_N_11	Not Reported	Hijra	Class 1–5	5,000 to 10,000 TK	Case Study Setting − 3	Hypertension
*PT_T_N_13*	Not Reported	Hijra	Class 1–5	Below 5,000 TK	Case Study Setting − 3	Not Reported
PT_T_N_14	18 to 25 years	Hijra	Class 6–8	Not Reported	Case Study Setting − 4	Not Reported
PT_T_N_20	26 to 30 years	Hijra	Never attended	Not Reported	Case Study Setting − 3	Not Reported
PT_T_N_21	Above 40 years	Hijra	Class 1–5	10,000 to 15,000 TK	Case Study Setting − 2	Hypertension
PT_T_N_22	Not Reported	Hijra	Not Reported	5,000 to 10,000 TK	Case Study Setting − 2	Not Reported
PT_T_N_23	18 to 25 years	Hijra	Class 9–10	Below 5,000 TK	Case Study Setting − 1	Not Reported
PT_T_N_24	Not Reported	Hijra	Class 1–5	5,000 to 10,000 TK	Case Study Setting − 1	Not Reported
PT_T_N_04	Above 40 years	Hijra	Never attended	Not Reported	Case Study Setting − 4	Both

#### Sampling of providers

There are significant differences in staff capacity between NGO clinics (case studies 1 to 4) and GoDs (case studies 5 and 6), with NGO clinics generally having larger staffing level (see Table 3). Each NGO clinic employs a total of 16 healthcare providers. For the case study sites, we selected one physician, one medical assistant (commonly referred to as a paramedic), and one pharmacist from each setting (*n* = 12). Similarly, each GoD employs six healthcare providers. For the GoD case study sites, we selected one physician, one SACMO, and one pharmacist from each site (*n* = 6). We specifically selected physicians, medical assistants, and pharmacists from each case study site as they are directly involved in service provision (See Table 4). Both male and female healthcare providers from each case study site participated in the interviews (*n* = 18). Prior to the interviews, providers were contacted via phone to schedule interviews at convenient times and locations for them.

**Table 3:** Staff capacity of primary healthcare facilities

Healthcare facilities	Type of Providers available in each case study setting	Number of providers allocated in each case study setting	Number of providers interviewed across the sampled case study settings
NGO Clinics (case study 1 to 4)	Physician	1	1
	Paramedic	4	1
	Pharmacist	1	1
	Counsellor	1	N/A
	Admin officer	1	N/A
	Management Information System (MIS) Officer	1	N/A
	Receptionist	1	N/A
	Lab technician	1	N/A
	Outreach staff − 4 (2 Family Welfare Assistants and 2 Service providers)	4	N/A
	Aya (Office support staff)	1	N/A
	Total number of healthcare providers allocated and interviewed across all NGO clinics	32	12
GoDs (case study 5 and 6)	Physician	1	1
	Sub Assistant Community Medical Officer (SACMO)	1	1
	Pharmacist	1	1
	Medical Lower Subordinate Staff (MLSS)	1	N/A
	Office support staff	1	N/A
	Night Guard	1	N/A
	Total number of healthcare providers allocated and interviewed across all GoDs	12	6

**Table 4:** Healthcare provider characteristics

Type of case study sites	Types of provider	Case	Case study setting
NGO Clinic	Physician	HP_A_d_01	Setting- 1
		HP_A_d_02	Setting- 2
		HP_B_d_01	Setting- 3
		HP_B_d_02	Setting- 4
GoD	Physician	HP_G_d_01	Setting- 5
		HP_G_d_02	Setting- 6
NGO Clinic	Pharmacist	HP_A_g_01	Setting- 1
		HP_A_g_02	Setting- 2
		HP_B_g_01	Setting- 3
		HP_B_g_02	Setting- 4
GoD	Pharmacist	HP_G_g_01	Setting- 5
		HP_G_g_02	Setting- 6
NGO Clinic	Paramedic	HP_A_e_01	Setting- 1
		HP_A_e_02	Setting- 2
		HP_B_e_01	Setting- 3
		HP_B_e_02	Setting- 4
GoD	SACMO	HP_G_h_01	Setting- 5
		HP_G_h_02	Setting- 6

### Data collection

Three male and female research assistants each with a postgraduate degree in public health, development studies, or anthropology, collected the data and were supervised by a senior researcher. They were all experienced in qualitative research, including conducting in-depth interviews, and proficient in NVivo, with a strong interest in urban health.

#### Patient interviews

The Commission for Social Determinants of Health (CSDH) Framework [24] was used to develop the patient topic guide, as it offers a comprehensive approach to understanding health by considering the broader social, economic, and cultural factors that shape health outcomes and healthcare experiences. Given the study's focus on patient experiences in PHCs, the framework illustrates how structural determinants such as socioeconomic status, gender, education, occupation and intermediary factors such as material and psychosocial conditions shape health behaviours and access.

The interview guide was initially pilot-tested with three patients (one male, one female, and one hijra) outside the case study sites. After the piloting, interview guides were revised to simplify the question wording. Interviews were then conducted between December 2022 to August 2023 at patients' homes, within the catchment area, and outside PHC facilities and no non-participants were present during the patient interviews. Each interview lasted for 15–30 min and after 30 patient interviews, the authors concluded that the data saturation point had been met and with no new information emerging. No repeat interviews were conducted and transcripts were not shared to participants for review.

#### Healthcare provider interviews

A separate topic guide was prepared for the healthcare providers based on WHO Health System Building Block framework [25] to ensure that questions were asked to explore their perspective on their primary care facility including service delivery, access to essential medicines, health workforce, financing, and governance. The topic guide was initially pilot-tested with three healthcare providers (one physician, one SACMO, and one pharmacist) outside the case study sites. Similar to patient topic guides, interview guides were revised after the piloting. The interviews were conducted between December 2022 to July 2023 and lasted 40–50 min. After 18 provider interviews, the authors concluded that the data saturation point had been met and no new information was emerging from the interviews. The interviews, primarily conducted within the facilities, may have influenced responses due to perceived surveillance or professional expectations.

No repeat interviews were conducted and transcripts were not returned to participants for review.

#### Observations

Each of the sites was visited by the research team to conduct structured observations of the facilities, equipment, recording and reporting mechanisms, and staff availability. The purpose of conducting the structured observations was to collect detailed notes and photographs to provide a more comprehensive understanding of the situation. Detailed notes were taken with photographs (with no staff or patients included). The photographs captured the facility's infrastructure, room layout, drug and equipment storage, medicine stock, and registration records, all of which were included in the analysis.

### Ethics approval and consent to participate

Ethical clearance for the study was given by the Bangladesh Medical and Research Council (BMRC) (BMRC/NREC/2019–2022/485) and the University of Leeds (No. MREC 21 − 008 CHORUS).

This study adhered to the ethical principles outlined in the World Medical Association (WMA) Declaration of Helsinki [26]. Prior to participation, all respondents were fully informed of the research objectives, procedures, potential risks, and benefits, both verbally and through a written information sheet. Participants were assured that their involvement was entirely voluntary and that they could withdraw at any time without consequence. Written informed consent was obtained from all participants before the interviews commenced. For individuals unable to sign, verbal and thumbprint consent were obtained, as per culturally appropriate and ethically approved practices. All participants were given the opportunity to ask questions and seek clarification before providing consent.

To ensure participant confidentiality and anonymity, unique identification codes were used in place of names, and all personal identifiers were excluded from transcripts and reporting. Data were handled with strict confidentiality throughout the study. Where participants agreed, interviews were audio recorded, otherwise, detailed verbatim field notes were taken in Bengali. Participation in audio recording was optional and based on explicit additional consent.

### Data analysis

The interviews were conducted in Bengali and later transcribed and translated verbatim into English for non-Bengali-speaking project partners. Data was analysed through the Framework Approach [27] following five stages: (1) familiarisation, (2) identifying a thematic framework, (3) coding, (4) charting, and (5) interpreting the data. During familiarisation, three researchers who had been involved in data collection read and re-read the transcripts to gain an in-depth understanding of the transcripts. A preliminary thematic framework was then developed from the CSDH and WHO Health System Building Blocks frameworks and refined inductively as new codes emerged from the data. Indexing and coding were performed in NVivo 12 by all three researchers independently, inter-coder reliability was assessed through comparison of codes and discrepancies were resolved through discussion until consensus was reached. Charting involved populating framework matrices (within NVivo and exported summary tables) to summarise data by case and theme, which supported cross-case comparison. Interpretations were undertaken iteratively to identify patterns, relationships and explanatory accounts, followed by synthesis into narrative summaries.

## Results

Our findings revealed discrepancies between the healthcare providers' and patients' perceptions of PHC services offered and operational effectiveness. These differences informed five key themes: (1) costs and perceptions of cost; (2) influence of medicine availability, equipment, and extended hours; (3) behaviour of healthcare providers; (4) maternal and child healthcare as the perceived primary role of PHCs; and (5) perceptions of NCDs.

This study found that providers' perceptions are shaped by specific characteristics of their healthcare facilities, including staffing levels, infrastructure quality, availability of medicines and equipment, and service hours. Patients' perceptions, on the other hand, are influenced by tangible aspects such as cleanliness, ambiance, accessibility, quality and availability of medical equipment. Equally important are the competence and attitudes of healthcare staff, as well as the efficiency of service delivery. These perceptions are further shaped by patients' socioeconomic status factors such as income, education, and insurance coverage influence their ability to access and afford care. Cultural and societal values also play a pivotal role in shaping how patients view healthcare. Beliefs about health and illness, and expectations regarding healthcare providers, influence their experiences. Furthermore, living and working conditions such as housing quality, employment status, and exposure to environmental hazards, affect both health outcomes and access to care. Behavioural factors including health-seeking behaviour, adherence to treatment, and lifestyle choices, as well as psychosocial elements such as stress, social support, and mental health, further affect how individuals engage with the healthcare system. Together, these elements form a complex web that collectively shapes community experiences and perceptions of healthcare quality.

Thus, only the proximal determinants of CDSH are reported within the themes. Together, these factors interact to shape patient experiences and community perceptions of healthcare quality. The WHO health systems building blocks provided the granular detail required to fully understand the health system element presented in the CSDH. This helped in identifying both patient and provider experiences of the health system, and these are presented below.

### Out-of-pocket costs

Within the domains of socio-economic position, findings from patient and provider interviews indicate that cost considerations, driven by rising out-of-pocket health expenditures, significantly affect the choice of PHC facilities. GoDs offer outpatient health services, consultations, limited diagnostic services and medications to all users, free of charge. As a result, many people seek services from these facilities because they offer comprehensive care without imposing financial burdens on vulnerable populations. In cotrast, NGO clinics provide healthcare services at subsidised rates to the general population, charging a nominal fee of 50 BDT (0.42 USD) for doctor consultations. Patients, however, are responsible for purchasing their medications. In addition, all NGO clinics offer discounts of 15% to 30% to all non-poor patients upon request, following approval from the project manager. Additionally, for the lower income group, a safety net provision known as *“red card”* ensures free access to consultations and medications for 30% of the total catchment area population. The allocation of red cards is based on economic evaluations conducted by NGO field workers through household surveys in the catchment areas of the clinics. These surveys assess factors within households such as the number of family members and earning members, number of rooms, assets, and monthly income. Households categorised as poor or ultra-poor based on this survey are then assessed for inclusion in the program, which provides every family member with free healthcare, including consultations, medications, lab tests, and even cesarean-sections. However, while the red card is intended to reduce financial barriers, we observed that not all eligible households could use it effectively. Misunderstandings about usage, household size, and recipients' limited education sometimes prevented households from benefiting fully. These access issues occur alongside other cost-related barriers such as the perceived high price of NCD medicines at NGO clinics, highlighting the multiple challenges patients face in accessing care.

*After being diagnosed as hypertensive*,* the doctor prescribed me Amlodipine which costs 210 BDT for each strip. Though I am advised to take it regularly*,* the high cost hinders me from doing so and visiting them again. – (Male*,* 42*,* NCD patient*,* Case Study Setting − 2*,* NGO Clinic*,* PT_M_N_18)*

Over half of the female participants reported facing financial barriers in accessing PHC services. While most were aware of nearby NGO clinics, nearly three-quarters of these women reported that they did not utilise the services primarily due to the associated out-of-pocket costs, which made consulting a professional physician difficult. These financial challenges were often compounded by additional expenses, such as transportation costs and lost wages. Similarly, among hijra participants who primarily earn their livelihood through small-scale contributions such as begging or collecting grains and money from households and public spaces, most reported that PHC services were largely unaffordable due to their low socio-economic status despite the clinics offering low-cost services. Over half of the hijra participants perceived PHC facilities as more expensive than local pharmacies, which led them to rely on cheaper, over-the-counter (OTC) medications rather than professional care.

*I’ve been suffering from high blood pressure for two years. When I feel sick*,* I go to the pharmacy to check my blood pressure and take primary remedies at home. I never go there as it takes money to consult a doctor from PHCs. (NCD Patient*,* Hijra*,* Didn’t disclose age*,* Case Study Setting-3*,* NGO Clinic*,* PT_T_N_11)*

*What could I do? I take a rest at home and buy napa (paracetamol) and other normal low-cost medicines. I cannot buy one file of medicine costing 500 BDT (4.19 USD) and visit a doctor who charges money. (Hijra*,* Didn’t disclose age*,* Case Study Setting-1*,* NGO Clinic*,* PT_T_N_24)*

## Availability of medicine, equipment, and extended hours

### Availability of medicines and equipment

Positioned within the CSDH framework, these findings reflect intermediary determinants related to the health system, specifically the availability of medicines, diagnostic resources, and service capacity. GoD staff reported that they have large medical supplies including a wide range of medicines used to treat common health conditions such as colds, fevers, diarrhoea, skin diseases, vitamin deficiencies, and joint pain. The available medications include antibiotics such as cefixime, penicillin, cephradine, flucloxacillin, amoxicillin, and clotrimazole. Moreover, they provide care for patients with NCDs, While these GoD lack specialist services and laboratory facilities, they do offer basic diagnostic services such as blood sugar testing and blood pressure measurement. Their medication stock is limited, consisting of Amlodipine for hypertension and Metformin for diabetes, along with antihistamines and calcium channel blockers. Notably, Amlodipine is more consistently available than Metformin. Due to the availability of medicines, patients from all categories frequently visit GoDs. This is particularly valuable given the high costs associated with obtaining these services elsewhere, despite the absence of laboratory services.

*About five or six months ago*,* I felt unwell with breathing problems*,* trouble moving*,* and a fear of collapsing. I went to see a doctor at this place*,* and he was surprised by my 160/170 high blood pressure. The doctor gave me some medicine for free. Now*,* whenever I’m sick*,* I go back to this facility.- (NCD Patient*,* Male*,* 47*,* Setting-5*,* GoD*,* PT_F_G_19)*

Moreover, patients who regularly visited these facilities, described a key reason for their repeated visits was the consistent availability of essential medicines.

*I’ve been to this facility before. Whenever I feel sick*,* I come to this centre because I live and work right next to it and medicines are free here.- (Male*,* 41*,* Setting-6*,* GoD*,* PT_M_G_01)*

In contrast, NGO clinics, perceived as focused on Maternity and neonatal child health (MNCH), attracting fewer patients for care related to NCDs and other non-MNCH conditions. This lower demand for non-MNCH care results in limited medication availability and drug shortages. Additionally, patients shared frustrating experiences where providers often refused to give them the necessary medications, even when in stock. In some cases, they were only given a limited supply for a short period, forcing them to purchase the medicines themselves most of the time. This situation left them feeling unsupported because they had expected more affordable options from these clinics but ultimately had to bear the financial burden themselves. Healthcare providers also acknowledge that their clinics' primary focus on maternal and child health limits their capacity to effectively manage NCDs. They have basic tools such as blood pressure monitors and glucometers, and they offer tests such as CBC, RBS, urine tests, HGPT, and lipid profiles. However, they lack essential NCD-related medications. Providers also highlight that their services are constrained by institutional priorities and resource limitations.

*Our project did not classify hypertension within their category. However*,* we do provide treatment if anyone comes with this issue but for services we do not have available*,* we refer patients elsewhere. If patients’ needs exceed our facility’s capabilities*,* how can we provide services beyond our current capacity!- (Physician*,* Setting-2*,* NGO Clinic*,* HP_A_d_02)*

### Operating hours and waiting times

Patients in urban slums reported a preference for healthcare facilities with extended operating hours and shorter waiting times, which correspond to the intermediary determinants of the CSDH framework, specifically the health system factors. This was particularly noted among male patients who work during the day and find it difficult to visit PHC facilities without affecting their schedules or income. Although GoDs operate for limited hours (9 am to 2 pm), majority of the patients appreciated their efficiency, noting short waiting times of 15–20 min per visit. This quick service contrasts with the long queues often seen in tertiary hospitals and other PHC facilities, allowing patients to seek care and still return to work within the same day. Patients also highlighted a preference for facilities with fewer crowds, which allows healthcare providers to spend more time with each patient, and for facilities with sufficient staff to provide comprehensive services while maintaining minimal waiting times.

*Tertiary hospitals are very crowded*,* and doctors get irritated and can’t give their full attention due to the enormous number of patients. It’s good that there is no waiting time*,* and the doctors do their job properly and it’s pleasant and peaceful here. - (NCD Patient*,* Female*,* 46*,* Setting-6*,* GoD*,* PT_F_G_17)*

In contrast, NGO clinics operate six days a week from 9 am to 4 pm, and from 9 am to 2 pm on Thursdays. Their strong focus on MNCH often results in long queues of women and children, limiting accessibility for male patients whose working hours coincide with clinic schedules. The perception that these facilities primarily serve women and children further discourages men from seeking care, contributing to lower attendance among men working outside the home. Additionally, patients reported difficulties accessing services due to early afternoon closures, particularly among those unable to take time off work. Some mentioned obtaining a ticket, but being unable to consult a doctor before the clinic closed.

*There are long queues and wait times to see doctors*,* and the hospital closes early in the afternoon. My wife once managed to get a ticket but couldn’t see the doctor as the hospital had already closed. After that experience*,* we never went there!- (NCD patient*,* Male*,* 36*,* Setting-4*,* NGO Clinic*,* PT_M_N_05)*

However, providers highlighted and agreed that given the greater number of men working outside the home, the facility's opening hours were a particular barrier for men working long hours.

*Since we provide services to the poor*,* impoverished*,* and middle class*,* maybe the male clients remain at their work that time*,* and as a result*,* we receive very few of them.- (Physician*,* Setting-1*,* NGO Clinic*,* HP_A_d_01)?*

## Behaviour of healthcare providers

Within the health system component of the CSDH framework, patients' experiences with healthcare providers' behaviour were critical in shaping their decisions to seek care at specific facilities. Providers across all the case study settings shared a strong commitment to delivering healthcare services with a focus on fairness, inclusivity, and non-discrimination. They emphasised their dedication to ensuring that every patient, regardless of their background whether social, cultural, gender, educational, or financial receives equal access to care and is treated with respect.

*Everyone is equal to us*,* whether he is rich or poor. In our centre*,* we uphold the policy that the first person to arrive will receive services first*,* regardless of who they are. (Pharmacist*,* Setting-2*,* NGO Clinic*,* HP_A_g_02)*

However, patients frequently reported that healthcare professionals at these facilities lack effective listening skills and exhibit rude behaviour, often displaying favouritism towards individuals from higher socio-economic backgrounds. Many individuals choose to avoid these institutions because they face discrimination based on gender and socio-economic status.

Additionally, patients with limited educational backgrounds often encounter difficulties in understanding the prescriptions at PHCs, which results in dissatisfaction. Furthermore, participants commented that the, pharmacies and private hospitals were more likely to provide detailed explanations of medical conditions and prescriptions, leading patients to opt for private facilities despite the higher costs. However, not all patients shared this view. Those living close to PHCs described having established good connections with facility staff, reporting that familiarity and consistency of care contributed to a more positive experience.

Several participants emphasised that trust and positive interactions with healthcare providers significantly shaped their experiences at PHC facilities. We found that patients felt more comfortable and confident in seeking care when providers took the time to explain diagnoses, listened attentively, and followed up on their concerns.

*As a person with disabilities and chronic conditions*,* I’ve always received incredible support at this centre. They’ve consistently provided me with medication and financial assistance*,* and the staff has been incredibly kind and helpful. - (Disabled NCD patient*,* Female*,* 40*,* Setting- 6. GoD*,* PT_F_G_16)*

While some patients considered PHC facilities a reliable source of care, marginalised populations particularly the hijra community, faced substantial barriers despite healthcare providers' stated commitment to inclusivity. The majority reported that providers hesitated to offer care due to concerns about negative reactions from other patients, and in some cases, they were denied entry, as facility staff feared that other patients would feel uncomfortable or leave. This stigma within the healthcare system discouraged hijras from seeking services at PHC facilities, leading many to rely on local pharmacies, where they experienced greater acceptance and could access care without facing humiliation or discrimination based on gender or sex. Participants also described challenges in accessing diagnostic and treatment services: physicians sometimes provided limited attention, laboratory staff were reluctant to handle tests, and hospital guards occasionally restricted entry. Furthermore, most participants reported that being referred to as “hijra” rather than by their personal name was perceived as disrespectful and caused discomfort. Consequently, hijras perceived that access to healthcare services at PHC facilities was influenced by financial status, employment, and gender, with hijras consistently receiving care last when other factors were equivalent. Many also relied on informal networks for livelihood, lived separately from their families due to social exclusion, and faced economic constraints that further limited access to healthcare services.

*During my visit to the NGO clinic*,* I was not permitted entry into the centre. Once they saw me*,* they inquired about my destination and informed me that it is forbidden to allow inside of a hijra according to the hospital authority instruction. (NCD Patient*,* Hijra*,* age not disclosed*,* Setting-3*,* NGO Clinic*,* PT_T_N_11)*

Physicians from NGO clinics acknowledged the challenges of providing care to hijras. They expressed concerns that admitting hijras to the facility might disrupt patient flow and create discomfort for other patients. Although they were willing to offer care to hijras, they emphasised the need for separate spaces to ensure the comfort of the general patient population.

*…if hijras enter the facility*,* people will get afraid. As a result*,* the patient flow and the quality of services will be decreased. We do not have any problem providing services to them but we need a separate space to provide services to them so that other patients do not get afraid of them.- (Physician*,* Settings-3*,* NGO Clinic*,* HP_B_d_01)*

## Maternal and child healthcare as the primary role of PHCs

Consistent with the intermediary determinants outlined in the CSDH framework, our findings indicate that although NGO clinics provide a primary care package that includes services for NCDs, patient visits for general ailments and NCD-related care remain relatively low. Healthcare providers noted that their services primarily focus on maternal and reproductive health, including antenatal care (ANC), postnatal care (PNC), neonatal care, immunisations, and family planning. Interviews with community members from the NGO clinic catchment areas revealed a widespread perception that these facilities prioritise MNCH, with limited capacity and resources for other health concerns. This perception was particularly common among male and Hijra participants. For some men, however, even the red card system was not enough to encourage them to use the NGO clinics due to the perception that these clinics were predominantly for women and children. We also found that the interview setting and prevailing gender norms influenced participant responses, shaping how patients perceived service availability and access. As one male NCD patient expressed-

*There’s a UPHC centre nearby*,* but I haven’t been there because I heard they only provide maternity services and immunisations.- (NCD Patient*,* Male*,* 48*,* Setting- 2*,* NGO Clinic*,* PT_M_N_18)*

*…. They said it was only for pregnant women and children*,* that’s why we don’t go there.- (Hijra*,* age not disclosed*,* Setting-3*,* NGO Clinic*,* PT_T_N_13)*

The perception that NGO clinics focus primarily on maternal and child health services was observed by both patients and healthcare providers. In one of our case study settings, we found that, while female patients make up the majority, there has been an increase in male patients with 327 male patients out of 1,321 visits in the previous month. Many healthcare providers acknowledged that their facilities tend to prioritise services related to maternal and child care often due to the high demand and available funding. They shared that many providers put up banners to show that the facilities welcomes everyone, hoping to encourage more men to visit. However, this belief persists among men, who often assume these facilities are exclusively for MNCH services, as well as within the hijra community, further limiting their engagement with these facilities.

## Perception of NCDs

Gender dynamics significantly influence health-seeking behaviours, particularly NCDs as misconceptions are prevalent among lower socioeconomic groups. Socio-cultural beliefs act as barriers to seeking care, particularly among men, as diseases such as diabetes and hypertension are perceived as illnesses affecting only wealthy people. This perception is often influenced by physically demanding jobs such as rickshaw pulling or construction work, leading them to believe they are resistant to these “rich people's” diseases. Additionally, some participants associate NCDs with divine punishment for past wrongdoings of rich people, consequently, dismissing the severeity of symptoms. Moreover, patients often underestimate the severity of NCDs, preferring rest and natural remedies over formal medical intervention. This differences in health-seeking behaviour, health literacy, and use of formal healthcare services reflect intermediary factors within the CSDH framework, including behavioural and psychosocial elements such as perceived susceptibility, illness beliefs, prioritisation of work over health, and interactions with the health system. Specifically, men's tendency to prioritise work due to socioeconomic responsibilities often leads to neglect of their health and delayed care-seeking.

Furthermore, men's prioritisation of work over their health due to socio-economical responsibilities leads to negligence in seeking medical care. In contrast, women are more concerned about NCDs and visit PHC facilities more frequently. We found that women from different social backgrounds were more aware of their health needs and followed their medications better when needed. They are more likely to visit healthcare facilities more often than men or other gender groups.

*…I was prescribed to take medicines for hypertension regularly*,* but taking medicines sometimes becomes an addiction for some people. My wife feels she’s about to die if she doesn’t have tablets. I am better than her without taking so I made the decision not to take the medication. If I could find a way to manage without it then why do I need to take medicine? (NCD Patient*,* Male*,* 50*,* Setting-6*,* GoD*,* PT_M_G_02)*

We also found that some patients particularly men, choose not to seek necessary healthcare services due to the belief that simple OTC medications such as paracetamol can cure all their ailments.

*I am aware of the nearby urban healthcare centre*,* but I never go there. When I get sick*,* I have a cup of tea sitting on my rickshaw and take a Napa Extra (Paracetamol). I don’t feel the need to visit the urban healthcare centre as it appears to prioritise profits over care.* - *(NCD patient*,* Male*,* 35*,* Setting-4*,* GoD*,* PT_M_N_05)*

## Discussion

We used a qualitative approach to explore patient and provider perspectives of PHC service provision in Dhaka to understand the factors influencing the choice of PHC facilities. The findings of this study both align with and diverge from the existing global literature on PHC utilisation. Consistent with research from other south asian cities, our results underscore that financial barriers and out-of-pocket rexpediture remain a critical obstacle to accessing PHC [28]. We found that both direct costs such as medicines, diagnostics, and consultation fees-and indirect costs, including travel and lost wages, as major deterrents to care-seeking. These findings reflect broader national trends in health financing. Between 2000 and 2020, the proportion of healthcare costs paid out-of-pocket in Bangladesh rose dramatically, from 11.54% to 74% [9]. This shift has placed an increasing financial burden on households, particularly among the urban poor, for whom even minimal expenses can delay or prevent access to essential care. In contrast, households with greater financial means face fewer constraints and are better positioned to access services across both public and private health sectors [28]. Targeted policy interventions are required to reduce out-of-pocket expenditures, particularly for low-income populations, through the implementation of health schemes that ensure free access to essential services.

Despite about 90% of communities having health facilities within two kilometers, marginalised groups, including the ultra-poor and third gender populations, face significant barriers [29]. As evidenced in our study, GoDs provide consultations and medications at no cost, whereas NGO clinics often impose user fees. In comparison, some countries have improved access to healthcare by offering community health insurance or subsidy programs [33], but in Dhaka, the “red card” system, led by NGO clinics, primarily benefits lower-middle-income groups. Unfortunately, it often fails to reach the poorest or most marginalised groups. This discrepancy contributes to inequities in service utilisation. Achieving harmonisation in Dhaka's urban health system is significantly challenged by institutional fragmentation and overlapping mandates. The split governance of NGO clinics under MoLGRD and government facilities under MoHFW leads to overlapping responsibilities, weak coordination, and limited accountability, undermining service delivery and patient experience. The Urban Health Strategy and evaluations of urban PHC in Bangladesh indicate that these structural issues hinder effective governance and oversight [30]. Addressing these issues through integrated planning, capacity strengthening of urban local bodies, and establishing stronger coordination mechanisms between ministries is essential to complement financial and access-focused interventions and improve the quality and equity of urban healthcare. However, NGO clinics, being primarily known for maternity services, further limit access for men and third gender communities. With limited public health care services available, the urban poor often rely on the private sector, such as pharmacies and drug shops, which can exacerbate exclusion of the ultra-poor and marginalised groups [31]. Evidence shows that, in Bangladesh, pharmacies are frequently the first and sometimes the only source of care, with over 80% of the population seeking care and OTC medicies from pharmacies. Out-of-pocket expenditure at pharmacies accounts for a significant portion of total health spending, highlighting the central role of informal providers in urban health-seeking behaviours [32]. Several low- and middle-income countries, including Ghana and Rwanda, have introduced social health insurance models to progress toward universal health coverage [33]. As healthcare costs represent a significant barrier that prevents individuals from converting their intention to seek care into actual utilisation of services [34], governments must strengthen health schemes and implement supportive regulatory, policy, and administrative frameworks for vulnerable populations [35].

Service quality is another key determinant influencing patients' trust and continued use of healthcare services. Positive interactions between patients and healthcare providers significantly shape perceptions of care [36]. Additionally, the physical environment, such as the availability of adequate space and efficient patient flow, also contributes to patient satisfaction and trust in the healthcare system [37] Consistent with these findings, our study revealed that patients who had stronger interpersonal relationships with providers reported more positive experiences. Participants felt more comfortable and confident in seeking care when providers clearly explained diagnoses, listened attentively, and followed up on their concerns. These observations highlight that trust-building and effective patient-provider communication are central to shaping patient experiences and encouraging service utilisation [38]. Beyond individual interactions, supportive social networks between patients and healthcare providers can further improve perceptions of PHC services by enhancing access, reducing vulnerability, and promoting equitable care. For example, evidence from Sub-Saharan Africa demonstrates that communities rely on such networks for financial, informational, and emotional support during health crises, which improves their ability to access care [39]. These networks buffer poverty's adverse effects through social integration and resource mobilisation, fostering inclusive healthcare delivery [40].

Moreover, we observed demographic differences between NGO clinics and urban dispensaries in Dhaka. NGO clinics are perceived as serving primarily women and children, while urban dispensaries serve more male patients despite being open to all genders.This pattern reflects both historical and structural factors. The bias toward MNCH in PHC facilities stems from historical global health priorities, particularly the Millennium Development Goals (MDGs), which emphasised reducing maternal and child mortality amid high burdens of infectious diseases [41]. Consequently, PHC services have been structured and resourced to address these priorities, shaping service availability and influencing patient demographics in urban settings [42]. At the same time, systemic barriers contribute to men's reliance on informal providers. Working-class men in economically disadvantaged communities often prefer pharmacies for basic care due to convenience, perceived affordability, and accessibility. To improve men's access to PHC services, targeted strategies such as health education, strengthened patient-provider communication through social networks, and male-focused community engagement initiatives are needed [39, 43].

### Strengths and limitations

This study has the following strengths. First, it considered and presented the dual perspective gained by interviewing both the patients and the healthcare providers. This approach allowed us to validate the information by contrasting and comparing the perspectives, rather than relying solely on data from the demand side (patients). Second, our sample included various types of healthcare providers from multiple layers of the organisation ensuring it represents the study population. Third, the inclusion of a diverse range of participants- male, female, and hijra individuals, including those with disabilities, across a wide age spectrum further enriched our study by providing diverse, varied and comprehensive insights.

Despite the study's strengths, this study has several limitations. First, the samples were restricted to DNCC and did not include all healthcare centres, which may limit its representativeness of the wider urban population. To mitigate this, a representative sample was taken from patients and providers across different socio-economic and gender groups. However, generalising these findings to other urban settings should be approached with caution.

Second, this study did not include any *hijra* participants visiting GoDs, limiting our ability to capture their perceptions and experiences regarding health-seeking behaviours. Third, the use of snowball and purposive sampling methods may introduce selection bias. Fourth, provider interviews were conducted on-site, sometimes with bystanders present. To minimise interruptions, researchers employed diversion techniques, however, responses may still have been influenced by perceived surveillance or professional expectations, representing a potential source of bias. Finally, the involvement of healthcare providers in participant selection may have also contributed to information bias, particularly given the suboptimal conditions for conducting interviews.

## Conclusion

While barriers to utilisation of government and non-government primary care are associated with limitations in services provided, patient perceptions of non-communicable diseases and primary care also play an important role in health-seeking behaviour. Our findings reaffirm the exclusion of men and hijras from MCH-focused PHCs, an outcome influenced not only by operating hours but also by the gendered nature of service design that prioritises maternal and child health. Strengthening trust and communication between providers and patients, and leveraging community and peer networks to enhance awareness and outreach, may help reduce this exclusion. These findings underscore that while global models provide valuable guidance, context-specific adaptations such as public-private partnerships, financial protection mechanisms, inclusive service redesign, and targeted community engagement are essential to address the intersecting inequities faced by urban populations in Dhaka city.

## Supplementary Information

Below is the link to the electronic supplementary material.

Supplementary Material 1

Supplementary Material 2

## Data Availability

Data for this work are held by the University of Leeds and ARK Foundation under managed access. Data are however, available from the corresponding author (mailto: nabilajahan.ku14@gmail.com) upon reasonable request.

## Abbreviations

PHC: Primary Health Care
LMIC: Low and Middle-Income Countries
UHC: Universal Health Coverage
NCD: Non-Communicable Disease
WHO: World Health Organization
GDP: Gross domestic product
HPNSP: Health, Population, and Nutrition Sector Program
NGO: Non-Government Organisation
MoLGRDC: Ministry of Local Government, Rural Development and Co-operatives
GoD: Government Outdoor Dispensary
CS: Civil Surgeon
MoHFW: Ministry of Health and Family Welfare
COREQ: Consolidated Criteria for Reporting Qualitative Research
DNCC: Dhaka North City Corporation
PHCC: Primary Health care Centres
CRHCC: Comprehensive Reproductive Health care Centres
UPHCSDP: Urban Primary Health Care Service Delivery Project
CDSH: Commission for Social Determinants of Health
SACMO: Sub-Assistant Community Medical Officer
MLSS: Medical Lower Subordinate Staff
MNCH: Maternity and neonatal child health
ANC: Antenatal Care
PNC: Postnatal Care
TT: Tetanus Toxoid
UPHCC: Upazilla Primary Health Care Crentre
OTC: Over-the-counter
MDGs: Millennium Development Goals

## References

[1] Alfaqeeh G, Cook EJ, Randhawa G, Ali N. Access and utilisation of primary health care services comparing urban and rural areas of Riyadh Providence, Kingdom of Saudi Arabia. BMC Health Serv Res. 2017;17:106. 28153002 10.1186/s12913-017-1983-z PMC5288856

[2] Michael GC, Aliyu I, Grema BA, Suleiman AK. Perception of factors influencing primary health-Care facility choice among National health insurance enrollees of a Northwest Nigerian hospital. J Patient Exp. 2019;6(3):247–52. 31535014 10.1177/2374373518790072 PMC6739678

[3] Allotey P, Davey T, Reidpath DD. NCDs in low and middle-income countries - assessing the capacity of health systems to respond to population needs. BMC Public Health. 2014;14(2):S1. 25082328 10.1186/1471-2458-14-S2-S1 PMC4120152

[4] Bangladesh - Urban population [Internet]. [cited 2025 Nov 14]. Available from: https://www.indexmundi.com/facts/bangladesh/urban-population?utm_source=chatgpt.com.

[5] WHO Bangladesh country cooperation strategy: 2020–2025 [Internet]. [cited 2024 Sep 22]. Available from: https://www.who.int/publications/i/item/9789290209478.

[6] World Bank [Internet]. [cited 2025 Feb 18]. The Continuum of Care for NCDs in Bangladesh: The Time to Act is NOW! Available from: https://documents.worldbank.org/en/publication/documents-reports/documentdetail/en/846731579019569888.

[7] World Bank Open Data [Internet]. 2024 [cited 2025 Feb 25]. World Bank Open Data. Available from: https://data.worldbank.org.

[8] Health Budget: ‘Increase allocation to combat NCDs’. The Daily Star [Internet]. 2024 Apr 7 [cited 2025 Feb 18]; Available from: https://www.thedailystar.net/news/bangladesh/news/health-budget-increase-allocation-combat-ncds-3583891.

[9] Khatun F, Saadat SY, Anika Ferdous Richi AF. Health budget of Bangladesh - optimising resources for improved health outcomes [Internet]. Centre for Policy Dialogue (CPD); 2024 Jul. Available from: https://cpd.org.bd/resources/2024/08/Health-Budget-of-Bangladesh-Optimising-Resources-for-Improved-Health-Outcomes.pdf.

[10] Islam MT, Bruce M, Alam K. Prevalence and correlates of health care utilization for non-communicable diseases in Bangladesh. BMC Health Services Research [Internet]. 2025 May 21 [cited 2025 Nov 2];25(1):736. Available from: 10.1186/s12913-025-12906-3. 10.1186/s12913-025-12906-3 PMC12093657 40400010

[11] Rawal LB, Kanda K, Biswas T, Tanim MI, Poudel P, Renzaho AMN, et al. Non-communicable disease (NCD) corners in public sector health facilities in Bangladesh: a qualitative study assessing challenges and opportunities for improving NCD services at the primary healthcare level. BMJ Open [Internet]. 2019 Oct [cited 2025 Feb 18];9(10):e029562. Available from: https://bmjopen.bmj.com/lookup/doi/10.1136/bmjopen-2019-029562. 10.1136/bmjopen-2019-029562 PMC6797278 31594874

[12] Islam K, Huque R, Saif-Ur-Rahman KM, Ehtesham Kabir ANM, Enayet Hussain AHM. Implementation status of non-communicable disease control program at primary health care level in Bangladesh: findings from a qualitative research. Public Health in Practice [Internet]. 2022 Jun [cited 2024 Mar 25];3:100271. Available from: https://linkinghub.elsevier.com/retrieve/pii/S2666535222000477. 10.1016/j.puhip.2022.100271 PMC9461504 36101774

[13] Shafique S, Bhattacharyya DS, Hossain MT, Hasan SM, Ahmed S, Islam R, et al. Strengthening health service delivery and governance through institutionalizing ’urban health Atlas’-A geo-referenced information communication and technology tool: lessons learned from an implementation research in three cities in Bangladesh. PLoS ONE. 2024;19(1):e0266581. 38271358 10.1371/journal.pone.0266581 PMC10810507

[14] Ali KJ, Noman ANK. Determinants of demand for health care in Bangladesh: an econometric analysis. 2013; Available from: https://www.researchgate.net/publication/280644503_Determinants_of_Demand_for_Health_Care_in_Bangladesh_An_Econometric_Analysis.

[15] Uribe MV, Gutierrez JC, Feil CS, Ramadan M, Sabina N, Hussain Z, et al. The primary health care system of bangladesh a primary health care performance initiative assessment [Internet]. World Bank Group; 2022 Aug [cited 2025 Feb 13]. Available from: https://documents1.worldbank.org/curated/en/099190002222311383/pdf/P159865039320203a097c301940d89c3473.pdf.

[16] Merry SP, Rohrer JE, Thacher TD, Summers MR, Alpern JD, Contino RW. Variables associated with utilization of a centralized medical post in the Andean community of Pampas Grande, Peru. J Rural Health. 2012;28(3):235–41. 22757947 10.1111/j.1748-0361.2011.00393.x

[17] Kalam A, Parvin S. Primary health care expectations, and reality of Bangladesh: a sociological analysis of the selected two rural areas. 2015.

[18] Höhn A, Gampe J, Lindahl-Jacobsen R, Christensen K, Oksuyzan A. Do men avoid seeking medical advice? A register-based analysis of gender-specific changes in primary healthcare use after first hospitalisation at ages 60 + in Denmark. J Epidemiol Community Health [Internet]. 2020 Jul [cited 2025 Feb 18];74(7):573–9. Available from: https://jech.bmj.com/lookup/doi/10.1136/jech-2019-213435. 10.1136/jech-2019-213435 PMC7337231 32303595

[19] The transgender healthcare gap: a pattern of stigma and struggle [Internet]. The Business Standard. 2024 [cited 2025 Nov 4]. Available from: https://www.tbsnews.net/thoughts/transgender-healthcare-gap-pattern-stigma-and-struggle-769374.

[20] Tong A, Sainsbury P, Craig J. Consolidated criteria for reporting qualitative research (COREQ): a 32-item checklist for interviews and focus groups. International Journal for Quality in Health Care [Internet]. 2007 Sep 16 [cited 2025 Apr 8];19(6):349–57. Available from: https://academic.oup.com/intqhc/article-lookup/doi/10.1093/intqhc/mzm042. 10.1093/intqhc/mzm042 17872937

[21] Crowe S, Cresswell K, Robertson A, Huby G, Avery A, Sheikh A. The case study approach. BMC Med Res Methodol. 2011;11:100. 21707982 10.1186/1471-2288-11-100 PMC3141799

[22] Yin RK. Enhancing the quality of case studies in health services research. Health Serv Res [Internet]. 1999 Dec [cited 2024 Sep 23];34(5 Pt 2):1209–24. Available from: https://www.ncbi.nlm.nih.gov/pmc/articles/PMC1089060/. PMC1089060 10591280

[23] Population & housing census 2022 preliminary report.pdf [Internet]. Bangladesh Bureau of Statistics; 2022 Aug. Available from: https://sid.portal.gov.bd/sites/default/files/files/sid.portal.gov.bd/publications/01ad1ffe_cfef_4811_af97_594b6c64d7c3/PHC_Preliminary_Report_(English)_August_2022.pdf.

[24] A conceptual framework for action on the social determinants of health [Internet]. [cited 2024 Sep 22]. Available from: https://www.who.int/publications/i/item/9789241500852.

[25] Everybody’s business -- strengthening health systems to improve health outcomes [Internet]. [cited 2024 Sep 22]. Available from: https://www.who.int/publications/i/item/everybody-s-business----strengthening-health-systems-to-improve-health-outcomes.

[26] WMA - The World Medical Association-WMA Declaration of Helsinki – Ethical Principles for Medical Research Involving Human Participants [Internet]. [cited 2025 Jul 6]. Available from: https://www.wma.net/policies-post/wma-declaration-of-helsinki/. 10.1001/jama.2024.21972 39425955

[27] Gale NK, Heath G, Cameron E, Rashid S, Redwood S. Using the framework method for the analysis of qualitative data in multi-disciplinary health research. BMC Med Res Methodol. 2013;13(1):117. 24047204 10.1186/1471-2288-13-117 PMC3848812

[28] Rao KD, Bairwa M, Mehta A, Hyat S, Ahmed R, Rajapaksa L, et al. Improving urban health through primary health care in south Asia. The Lancet Global Health [Internet]. 2024 Oct [cited 2025 Apr 30];12(10):e1720–9. Available from: https://linkinghub.elsevier.com/retrieve/pii/S2214109X24001219. 10.1016/S2214-109X(24)00121-9 39178875

[29] National Institute of Population Research and Training (NIPORT). Urban Health Survey 2021 [Internet]. 2022 Mar [cited 2024 Nov 20]. Available from: https://niport.portal.gov.bd/sites/default/files/files/niport.portal.gov.bd/publications/66c4accd_4c6a_4aab_902c_781a77aa8768/2023-01-30-06-04-9310fc1f3902cdd03884124c600ddc8d.pdf.

[30] Health services division, ministry of health and family welfare, government of the People’s Republic of Bangladesh. National urban health strategy 2020. February; 2020.

[31] Roy S, Sowgat T. Dhaka: diverse, dense, and damaged neighbourhoods and the impacts of unplanned urbanisation. 2020; Available from: https://www.centreforsustainablecities.ac.uk/wp-content/uploads/2020/10/SHLC_Research_Summary_08_DHAKA.pdf.

[32] Ahmed SM, Naher N, Hossain T, Rawal LB. Exploring the status of retail private drug shops in Bangladesh and action points for developing an accredited drug shop model: a facility based cross-sectional study. J Pharmaceut Policy Practice [Internet]. 2017 Dec 31 [cited 2025 Nov 4];10(1):21. Available from: https://www.tandfonline.com/doi/abs/10.1186/s40545-017-0108-8. 10.1186/s40545-017-0108-8 PMC5506600 28702204

[33] George Anarwat S. Health insurance for economically disadvantaged people in LMICs: What are the Best Options? Isabel Tavares A, editor. Health Insurance [Internet]. 2022 Oct 19 [cited 2024 Nov 24]; Available from: https://www.intechopen.com/chapters/82873.

[34] Park JE, Kibe P, Yeboah G, Oyebode O, Harris B, Ajisola MM, et al. Factors associated with accessing and utilisation of healthcare and provision of health services for residents of slums in low and middle-income countries: a scoping review of recent literature. BMJ Open [Internet]. 2022 May [cited 2024 Nov 24];12(5):e055415. Available from: https://bmjopen.bmj.com/lookup/doi/10.1136/bmjopen-2021-055415. 10.1136/bmjopen-2021-055415 PMC9125718 35613790

[35] Shah A, Lemma S, Tao C, Wong J. The role of health policy and systems in the uptake of community-based health insurance schemes in low- and middle-income countries: a narrative review. Health Serv Insights [Internet]. 2023 Jan [cited 2024 Nov 24];16:11786329231172675. Available from: https://journals.sagepub.com/doi/10.1177/11786329231172675. 10.1177/11786329231172675 PMC10155025 37153878

[36] Shie AJ, Huang YF, Li GY, Lyu WY, Yang M, Dai YY, et al. Exploring the relationship between hospital service quality, patient trust, and loyalty from a service encounter perspective in elderly with chronic diseases. Front Public Health [Internet]. 2022 May 25 [cited 2024 Nov 21];10:876266. Available from: https://www.ncbi.nlm.nih.gov/pmc/articles/PMC9174694/. 10.3389/fpubh.2022.876266 PMC9174694 35692341

[37] Public trust in. physicians: empirical analysis of patient-related factors affecting trust in physicians in China | BMC Primary Care | Full Text [Internet]. [cited 2024 Nov 21]. Available from: https://bmcprimcare.biomedcentral.com/articles/10.1186/s12875-022-01832-6. 10.1186/s12875-022-01832-6 PMC9427175 36042408

[38] Zakaria M, Mazumder S, Faisal HM, Zannat R, Haque MR, Afrin T, et al. Physician communication behaviors on patient satisfaction in primary care medical settings in Bangladesh. J Prim Care Community Health [Internet]. 2024 Sep 26 [cited 2025 Nov 3];15:21501319241277396. Available from: https://pmc.ncbi.nlm.nih.gov/articles/PMC11452860/. 10.1177/21501319241277396 PMC11452860 39327849

[39] Amoah PA, Edusei J, Amuzu D. Social networks and health: understanding the nuances of healthcare access between urban and rural populations. IJERPH [Internet]. 2018 May 13 [cited 2025 Mar 26];15(5):973. Available from: https://www.mdpi.com/1660-4601/15/5/973. 10.3390/ijerph15050973 PMC5982012 29757256

[40] Keim-Klärner S, Adebahr P, Brandt S, Gamper M, Klärner A, Knabe A, et al. Social inequality, social networks, and health: a scoping review of research on health inequalities from a social network perspective. Int J Equity Health [Internet]. 2023 Apr 25 [cited 2025 Mar 26];22(1):74. Available from: https://equityhealthj.biomedcentral.com/articles/10.1186/s12939-023-01876-9. 10.1186/s12939-023-01876-9 PMC10131340 37098617

[41] Roy A, Shengelia L. An analysis on maternal healthcare situation in Bangladesh: a review. Diversity & Equality in Health and Care [Internet]. [cited 2025 Nov 4];13(5):0–0. Available from: https://www.primescholars.com/.

[42] Khalequzzaman Md, Chiang C, Hoque BA, Choudhury SR, Nizam S, Yatsuya H, et al. Population profile and residential environment of an urban poor community in Dhaka, Bangladesh. Environ Health Prev Med [Internet]. 2017 Dec [cited 2024 Nov 20];22(1):1. Available from: https://environhealthprevmed.biomedcentral.com/articles/10.1186/s12199-017-0610-2. 10.1186/s12199-017-0610-2 PMC5661908 29165111

[43] Leal Borges CC, De Sousa AR, Araújo IFM, Ortiz Rodríguez JE, Escobar OJV, Martins RD, et al. A scoping review of men’s health situation in primary health care. TONURSJ [Internet]. 2021 Dec 15 [cited 2025 Apr 30];15(1):412–21. Available from: https://opennursingjournal.com/VOLUME/15/PAGE/412/.

