# Supplementary material for: Patient and provider perspectives of pluralistic primary care services in urban Bangladesh: a qualitative study
Source: BMC Health Serv Res. 2026 Feb 14;26:384. doi: 10.1186/s12913-026-14106-z (PMC13011357; doi:10.1186/s12913-026-14106-z)
Supplement: Supplementary file 1 — Supplementary Material 1 [file 12913_2026_14106_MOESM1_ESM.pdf]

Strengthening the urban primary health care system to deliver essential NCD care to urban poor: An Implementation Research

Topic Guide for Health Care Providers

A. Service Delivery

1. Patient load on a regular day?
    a. Factors affecting patient load
    b. What types of patients visit?
    c. What care do the majority of patients seek?
    d. Age range?
    e. Gender?
    f. Mentally / physically disabled?
2. Are all patients screened for NCDs?
    a. If yes, which NCDs are they screened for?
    b. If no, what factors determine the necessity to screen a patient for NCD?
3. Are NCD patients listed?
    a. Where, how, and who is responsible?
4. Are patients with a risk of developing NCD listed?
    a. Where, how, and who is responsible?
5. Scene 1: Patient is screened and is not found to have any NCD. What happens next?
    a. If follow-up is advised, do they follow?
        i. If yes, how often?
        ii. If not, why not?
    b. Are family members counselled?
6. Scene 2: Patient is screened and is found to have an NCD. What happens next?
    a. If follow-up is advised, do they follow?
        i. If yes, how often?
        ii. If not, why not?
        iii. Are family members counselled?
7. Scene 3: The Patient is an NCD patient. What happens next?
    a. If follow-up is advised, do they follow?
        i. If yes, how often?
        ii. If not, why not?
        iii. Are family members counselled?
8. Scene 4: Patient with Comorbidity, what happens next?
    a. If follow-up is advised, do they follow?
        i. If yes, how often?
        ii. If not, why not?
        iii. Are family members counselled?
9. What is the referral mechanism for NCD care?

B. Urban poor

1. Does the system identify the urban poor and/or marginalised population?
    a. If yes, how, criteria?
    b. If yes, are they documented?
        i. Person/ministry responsible?
        ii. Where?
        iii. Is the list updated? If yes, frequency?
        iv. Maintenance of the list?
    c. If yes, how are their health care needs met, especially when it comes to NCD?
2. Any existing guidelines for NCD care of marginalised people / urban poor?
3. Availability of free or subsidised treatment for the urban poor or marginalised community, especially for NCD?
    a. If yes, any specific NCD?
    b. If yes, what factors are taken into account while providing free or subsidised treatment?
    c. Documentation or records of the above – Available? If yes, where? Who maintains? How are they maintained?

C. Human resources

1. Are the manpower pertaining to NCD care recorded?
    a. If yes, how and who is responsible?
2. Transfer of personnel to another department/health care facility?
3. Substitute in case of the absence of allocated personnel?
4. Are existing skills and knowledge of NCD care among primary health care providers assessed?
    a. If yes, how?
5. IF assessed, who assesses, and is the assessment recorded?
    a. If yes, how?
6. IF assessed, which NCDs are covered?
7. Existence of NCD training programmes
    a. Participants –
        i. Who?
        ii. From where?
        iii. Only doctors or nurses, too?
    b. Topics?
    c. Who conducts, who funds?
    d. Basic and refresher?
    e. Frequency
    f. Length of training?
    g. Location – Central or periphery
    h. Incentives?

        i. Challenges in training
        j. Existence of NCD control guidelines?
        k. Updates in NCD care

D. Meedicine and Equipment

1. Are the medicine and equipment pertaining to NCD care recorded?
    a. If yes, how and who is responsible?
    b. If not, why not?
2. NCD medicine and equipment storage
    a. How?
    b. Where?
    c. Responsible person?
    d. Examining the calibration of equipment?
        i. Who?
        ii. How?
        iii. Frequency?
        iv. Documentation?
        v. Accountability?
3. Distribution of medicine and equipment related to NCD
    a. Who?
    b. How?
    c. Frequency?
    d. Accountability?
    e. Documentation?
4. Challenges in storing NCD medicine and equipment?
    a. If yes, what are they?
    b. How are they handled?

E. Monitoring and evaluation

1. What is the monitoring mechanism?
    a. Any supporting documents or documenting system?
2. What is the flow of reporting, especially for NCD care?
3. Existing reporting framework for NCD care?
4. Supervision visits?
    a. Who?
    b. If yes, what is supervised and how?
        i. Service?
        ii. Equipment
            - Calibration and storage?
    c. Frequency?
    d. Documentation – how? Who? Where?
5. Monitoring and supervision reports
    a. Who?

b. To Whom?
c. Frequency?
d. Templates?

F. Intersectionality

1. Are you familiar with the term “Intersectionality”?

If YES,

a) Where did you learn this term from?
b) Could you explain what it means to you?
c) Can you relate this concept to your clinical practice/healthcare profession?

2. How likely are people from the third gender to face comparatively more challenges at urban health facilities while seeking NCD care?

3. Do the urban facilities provide the same treatment for major NCDs to people from all socioeconomic classes? Is it possible that the treatment protocol or prescriptions will be different for the poorer groups than those of the wealthier? Why?

4. Across all genders and socioeconomic classes, does the education level of a patient impact their NCD treatment? For example, is it easier for the healthcare providers and managers to provide efficient services to a highly educated woman with Diabetes Mellitus than to a less educated woman?

G. Recommendations
