# Supplementary material for: Patient and provider perspectives of pluralistic primary care services in urban Bangladesh: a qualitative study
Source: BMC Health Serv Res. 2026 Feb 14;26:384. doi: 10.1186/s12913-026-14106-z (PMC13011357; doi:10.1186/s12913-026-14106-z)
Supplement: Supplementary file 2 — Supplementary Material 2 [file 12913_2026_14106_MOESM2_ESM.pdf]

Strengthening urban primary health care system to deliver essential NCD care to urban poor: An Implementation Research

Topic Guide for Patients

Demographics:

1. Age
2. Sex
3. Marital Status
4. Level of education
5. Monthly family income
6. Do you consider yourself a disabled person?

Healthcare Seeking Behaviour:

1. Tell me about the last time you were ill
2. Did you go to any treatment or seek advice from anyone? Where did you go?
3. What kind of services do you usually seek?
4. How many times have you visited/how often do you visit? Reasons for visiting?
5. Do you suffer from any NCD (Hypertension, diabetes, CKD, CVD, etc.)?

   If yes, tell me about your experience with diabetes, hypertension, or heart disease.
   If no, can you tell us about the risk factors (tobacco use, diet, exercise), and if you have ever considered having a check-up to be screened?

   a) Who diagnosed you? Where? Have you ever gone to the urban dispensaries for NCD?
   b) Why did you consider getting screened? What symptoms did you experience before NCD screening?
   c) Did you get yourself checked up immediately after experiencing symptoms/feeling the necessity of it?
      If not, how long did you wait and why?
   d) Which NCD disease are you suffering from?
   e) How long has it been since you were diagnosed with the NCD(s)?
   f) How often do you visit the facility for follow-up?
   g) Have you ever been referred to another hospital/specialist?
   h) Where do you go for the diagnostic tests?
   i) Are you under any medication for the NCD(s)?
      If yes, do you take your medication regularly? Have you got any type of counselling from the healthcare facilities?
      - If any, are you maintaining/following the guidelines provided by the HP to control NCDs?
      - *If yes,* is it helping you to control NCDs?
   j) Have you ever been screened for any NCD (i.e., checking blood pressure, sugar, urine tests, etc.)?
6. Do you go to any other healthcare provider for NCD care? Where do you usually go?

Experience with the healthcare providers at the urban healthcare facilities

1. Have you heard about the urban dispensaries?
    a) If yes, have you ever visited there?
    b) Tell me about your experience while visiting.
    c) Describe what happened when you first arrived at the clinic. Who did you see? What did they ask you? What did you think about what they told you?
    d) If not, why didn't you visit there?
    If yes, reason for the visit?

2. Interaction with doctors
    a) Taking medical history, giving enough time to explain
    b) Behaviour of the doctor
    c) Did they explain the health conditions in detail?
    d) Did they explain the treatment process well? Did they notify you about side effects of or cautions regarding the prescribed medications?
    e) Did they provide any preventive advice (i.e., lifestyle modification, regular screening/check-up, etc.)?

3. Interaction with the pharmacist
    a. Behaviour
    b. Did they explain the necessary instructions carefully?
    c. Do they take the necessary measurements carefully? (Patients' height, weight, BP, etc.)
    d. If/when approached with questions related to the disease and treatment, do they answer clearly and elaborately?

4. Interaction with SACMOs
    a. Behaviour and approach of sample collectors
    b. How caring and careful were they while collecting the samples?

5. Do you find the NCD care accessible in terms of:
    a) Ease of accessing the doctors and nurses
    b) Availing prescribed medication
    c) Availing emergency medical help if necessary
    d) Diagnosis

Recommendations

1. Ideally, how would you like the services to be at the GOD?
2. How can they best help you manage your diabetes, hypertension, etc.?
3. What changes would need to happen at the GOD to make it a good location/place for you to get treatment/support to manage your diabetes/hypertension, etc?
